# Context of Breastfeeding among Latina Mothers using a Social-ecological Approach: An Exploratory Study

**Published:** 2018-11-23

**Authors:** Alice Ma, Elisa A. Merçon-Vargas, Brittany D. Chambers, Monde Nyambe, Tiffany A. Williams

**Affiliations:** 1 Department of Applied Health, Southern Illinois University Edwardsville, Edwardsville, IL, 62025-1126, USA; 2 Department of Social and Developmental Psychology, Federal University of Espírito Santo, Vitória, Espírito Santo, 29075-073, Brazil; 3 Department of Epidemiology and Biostatistics, UCSF Preterm Birth Initiative, University of California San Francisco, San Francisco, CA, 94158, USA

**Keywords:** breastfeeding, Latina mothers, culture, qualitative, social-ecological model

## Abstract

**Background:**

Emerging research has begun to examine the breastfeeding experiences among racial/ethnic minority women. However, limited research to date has explored the potential factors that impact Latina mothers’ breastfeeding through a multi-level lens. We examined the context of breastfeeding among Latina mothers in an exploratory study.

**Methods:**

We conducted semi-structured interviews with a convenience sample of 9 Latina mothers. Guided by the social-ecological model, thematic content analysis was used.

**Results:**

Latina mothers described individual- (e.g., knowledge of breastfeeding), interpersonal- (e.g., social support and norms), institutional- (e.g., healthcare system), and community-level (e.g., cultural norms) influences on their breastfeeding. Mothers recommended provision of bilingual and bicultural health professionals, information on US breastfeeding norms, and Latino-friendly informational materials in other languages.

**Conclusions:**

Considering the multi-level factors that shape Latina mothers’ breastfeeding is essential to develop and implement culturally tailored initiatives and facilitate access to breastfeeding support to improve maternal and infant health.

## Introduction

In accordance with recommendations from the Global Strategy on Infant and Young Child Feeding [1] and the American Academy of Pediatrics [2], breastfeeding is the optimal feeding standard for infant nutrition [3]. Despite the benefits of breastfeeding (e.g., reduced risk of post-partum hemorrhage) and high initiation rates (76.5%) among new mothers in the United States (US), overall rates for continued breastfeeding remain low (49.0% at 6 months versus 27.0% at 12 months), particularly for certain racial/ethnic groups [4,5]. Although Latina mothers’ breastfeeding initiation rates are typically higher compared to other racial/ethnic groups (80.0% versus 75.2% among White and 58.9% among Black/African American mothers) [6], their exclusive breastfeeding rates tend to be lower and formula supplementation rates higher compared to other racial/ethnic groups, which can reduce breastfeeding duration [7–9]. These rates challenge Healthy People 2020’s national goals for breastfeeding (e.g., increasing the proportion of infants who are breastfed and reducing the proportion who receive formula supplementation), which suggests barriers to breastfeeding that may be uniquely experienced by some racial/ethnic minority women (e.g., English-language abilities) [10,11]. To ensure that minority women, such as Latina mothers, are supported in their breastfeeding during this critical time, it is important to identify opportunities to reduce barriers and increase facilitators to breastfeeding.

Latina mothers’ breastfeeding practices are impacted by a variety of factors, including cultural norms, social support, and access to culturally congruent care [9,11–14]. These factors may differentially impact breastfeeding behaviors on a continuum, with some breastfeeding more due to a more supportive context (e.g., access to breastfeeding support services) and others breastfeeding less due to a more restrictive context (e.g., conflicting cultural breastfeeding norms) [9,11,13]. The interplay of these factors (e.g., culture and social support) that shape Latina mothers’ breastfeeding suggest the importance of examining them through a multi-level lens [15,16]. Although emerging research has begun to examine the breastfeeding experiences among racial/ethnic minority women [16], to our knowledge, no research has yet explored the potential factors that impact Latina mothers’ breastfeeding practices through a multilevel lens. Existing research with Latina mothers has primarily focused on individual- and interpersonal-level examinations of their breastfeeding experiences (e.g., knowledge of breastfeeding and social and cultural perceptions) [9,13,17].

Accordingly, we examined the context of breastfeeding among a small sample of Latina mothers using the social-ecological model in an exploratory study.

## Materials and Methods

### Participants and data collection:

We conducted an exploratory study with nine Latina mothers who resided in a diverse, mid-sized city located in the southeastern US. Inclusion criteria included: (1) self-identified as Latina or Hispanic; (2) age 18 years or older; (3) have at least one child of any age; (4) have initiated breastfeeding at any time (i.e., feeding breast milk via the breast directly and/or expressing milk); (5) be able to speak English; and (6) willingness to share their breastfeeding experiences. After each interview, we asked the participant to suggest additional contacts who met the inclusion criteria. Data collection occurred between March and April 2014. Participants were selected via snowball and criterion sampling. We also recruited participants through flyers, personal contacts, social media posts to local groups, e-mails, and phone calls. Recruitment ceased when similar themes emerged in the interviews and a variety of nationalities were represented. The research team determined this point to be data and sampling saturation. The Institutional Review Boards at the University of North Carolina at Greensboro and Southern Illinois University Edwardsville provided human subject review and study oversight (#14-0097 and #18-0122-1C).

We maintained a detailed audit trail to document our study procedures. After participants gave informed consent, we conducted interviews using a semi-structured guide in a setting and time convenient for them. We used open-ended questions to elicit participants’ breastfeeding experiences and probes to prompt elaboration. All interviews were audio-recorded and transcribed verbatim by the researcher present for the interview. The average interview length was approximately 25 minutes. Table 1 presents sample interview questions. No compensation was provided to participants.

coincides with the first letter of her self-identified native country (e.g., Barbara’s native country is Brazil). Data were entered and managed using Microsoft Word.

## Results

Nine Latina mothers participated in the study; their demographic characteristics are presented in Table 2. They identified with a variety of nationalities, including Brazil (n=3), the Dominican Republic (n=l), Mexico (n=3), and Puerto Rico (n=2). The average age was 36 years (range: 22–49 years). Slightly more mothers reported a long breastfeeding duration (n=4) than those who reported a short duration (n=3).

The Latina mothers identified a variety of factors as salient in their breastfeeding experiences across the social-ecological model [18]. Overall, the mothers described navigating breastfeeding in the US as “very complicated” (Barbara, Brazil), with one mother noting the continual challenges of breastfeeding in the US: “Well, I would assume here [in the US], it [breastfeeding] would be getting easier, but apparently it’s not” (Maria, Mexico).

### Individual:

Knowledge of breastfeeding. The Latina mothers shared basic knowledge of breastfeeding. Many highlighted the importance of breastfeeding and the benefits associated with it: “The kids will be healthier and more immune to diseases from the outside when breastfeeding” (Patricia, Puerto Rico). Several mothers stressed that

### Analysis:

Guided by the social-ecological model [18] and interview questions, we used content analysis to interpret the breastfeeding experiences described by Latina mothers [19]. We developed a priori codes (e.g., social support) based on the literature. We added and compared emergent codes (e.g., healthcare) based on the interview transcripts. The finalized coding structure was then applied to the interview transcripts; a coding glossary and hierarchical coding chart facilitated this process. Coding discrepancies were resolved by discussion. Exemplar quotations were used from each participant to identify patterns, then common themes were generated based on these codes and patterns. We analyzed and discussed interviews as they occurred, individually and in groups, and compared them to confirm or disconfirm emerging themes. The quotations presented reflect main themes from the participants. Participant information was deidentified with pseudonyms: the first letter of each pseudonym breast milk provided the best nutrients (e.g., vitamins) and contributed to the immunization and protection of the child (e.g., antibodies) in the short- and long-term. Further, mothers reported that breastfeeding contributed to their physical and psychological well-being, including nurturing the bond with their child. They also noted that breastfeeding helped them lose the weight gained during pregnancy. The mothers’ knowledge of breastfeeding and its benefits are summarized by Bruna (Brazil): “So for the mother…It [breastfeeding] is good to create a bond with the child, it is good to slim, it is good to the self-esteem, it is good because it is delightful…”

### Interpersonal:

Social support and norms. Many Latina mothers reported having a strong support system from family members and friends. To some extent, this support influenced their decision to breastfeed. Several mothers noted that almost everyone they knew, either growing up or as adults, breastfed their children: “Culturally, I was raised seeing everybody in my family or in the community breastfeeding” (Dolores, Dominican Republic). Friends and family often validated the importance of breastfeeding - many of whom shared the mothers’ cultural background and were proponents of breastfeeding. Paloma (Puerto Rico) described, “My mother-in-law and my mom were always like, ‘You’re gonna breastfeed, right? That’s important, you have to, you have to! ‘”

Additionally, some mothers noted the lack of information on US breastfeeding norms as a critical barrier. For instance, Bianca (Brazil) expressed it was through her friends that she was alerted to the taboos of public breastfeeding in the US: “But if my Brazilian friends had not told me, I wouldn’t know…And maybe I would get to an embarrassing situation.” In this case, friends were a more important source of information on US cultural breastfeeding practices than healthcare professionals.

However, when some mothers experienced unsuccessful or complicated breastfeeding, they reported psychological distress, such as disappointment, sadness, and even grief. When Maria (Mexico) was not able to breastfeed due to latching difficulties, she recalled feeling “really, really, really sad and really mourned not being able to nurse him.” This internal pressure and expectation to breastfeed, and at times subsequent distress, illuminate the psychological challenges and the need for social support, particularly emotional support, when mothers breastfeed.

### Institutional:

Healthcare. Although breastfeeding was widely accepted across the Latina mothers, they noted a dearth of Latino-friendly resources in the healthcare system to access breastfeeding information. Despite some mothers enjoying the “many courses” (Bianca, Brazil) and other hospital support services, such as postpartum in-home support, others reported language difficulties, including issues finding bilingual health professionals. Patricia (Puerto Rico) noted, “I don’t know if they have enough material in Spanish and that would help because…they come here [US], it’s a culture mix, and they got lost in the culture and sometimes they don’t know.”

Notably, Paloma (Puerto Rico) described “a lot of prejudice” witnessed at a clinic appointment with her Latina friend. She believed her friend experienced poor quality service due to her friend’s limited English-speaking abilities: “They were nicer to me than her [my friend]. And I didn’t like that…I think the girl was a little bit rude with her. Maybe she got frustrated because she [my friend] didn’t understood her or something.”

### Community:

Cultural norms. The Latina mothers noted the cultural differences in breastfeeding between their native countries and the US. They emphasized breastfeeding in their culture as natural, therefore public breastfeeding was not an issue, as explained by Barbara (Brazil) : “I think in Brazil, breastfeeding…it is much more necessary. We see that as, it is basic…You can breastfeed your child in public, here you cannot.” Oftentimes, the mothers perceived breastfeeding in the US as “not acceptable” (Dolores, Dominican Republic), with typically longer breastfeeding durations in their native country than in the US.

### Recommendations:

The Latina mothers emphasized the need for more breastfeeding support services and awareness, particularly for bilingual and bicultural healthcare professionals and resources: “Maybe doing more like classes, workshops about these things [breastfeeding]…because the Hispanic—most of the time, they really don’t know. It’s not that they don’t want to, it’s that they don’t really know, they don’t have the information…So it will be more Spanish material and more maybe workshops…” (Patricia, Puerto Rico).

Although healthcare clinics provided some Latino-friendly informational materials (e.g., pamphlets), Maria (Mexico) noted the dearth of Spanish-speaking local healthcare professionals -“There’s just not very many bilingual health professionals here” -which can challenge connection to a healthcare provider and limit breastfeeding education for Latina mothers. Dolores (Dominican Republic) also recommended the inclusion of health educational materials in Spanish and other languages. One recommendation to help disseminate breastfeeding information to Latina families (not only mothers) was to implement a community breastfeeding event: “I think it would be awesome if…[there is] a breastfeeding day and kind-of do like an education or awareness…that help people in the community to become interested and have more information out, so it doesn’t become so much it’s just—like a women thing” (Dolores, Dominican Republic).

## Discussion

The findings from this exploratory study highlight the breastfeeding practices of a small sample of Latina mothers residing in the southeastern US. Guided by the social-ecological model [18], the mothers identified multi-level factors salient in their breastfeeding experiences, including knowledge of breastfeeding, social support and norms, the healthcare system, and cultural norms. Despite the limited sample, our findings identified common themes related to the importance of breastfeeding that emphasized personal and social aspects, including the benefits for the infant and mother. Their breastfeeding knowledge aligns with established literature on the benefits of breastfeeding, including bonding opportunities and reduced infant health risks [20], However, given the lower rates of exclusive breastfeeding and higher rates of formula supplementation compared to other racial/ethnic groups [7–9], knowledge of breastfeeding alone may be insufficient to encourage breastfeeding among Latina mothers [13]. Our findings support emerging research that considers multi-level factors that impact breastfeeding; this approach can aid in comprehensively understanding Latina mothers’ breastfeeding behaviors and identifying opportunities for solutions to support them [15,21].

The Latina mothers often situated their breastfeeding practices in terms of their culture [9,11,22,23]. The mothers’ support system shared similar perspectives with them on the importance of breastfeeding, yet their cultural beliefs - and thereby pressures - to breastfeed were at times challenging. Although a supportive environment is critical to mothers’ relationship with breastfeeding [23–25], they may also need to be supported when breastfeeding difficulties arise. These mothers tended to experience psychological distress, suggesting a need for comprehensive support systems that are culturally congruent, particularly for those who may experience multiple, co-occurring difficulties [26,27].

Notably, our findings illuminate a potential dearth in healthcare support for Latina mothers. Some mothers described challenges in navigating the US healthcare system, such as language barriers and discrimination, which may suggest critical issues in accessing some breastfeeding support services [28]. Some mothers noted the lack of information on US cultural norms, which suggests the need for more Latino-friendly informational support on cultural norms. Developing culturally tailored efforts and enhancing diversity training among healthcare professionals may be critical to support care for Latina mothers [17]. Given that racial/ethnic disparities remain in breastfeeding rates and practices [11], it is essential to understand and enhance culturally congruent care to ensure high quality services for vulnerable mothers [29].

Although this study included participants from a variety of nationalities, our small sample is unlikely to represent all Latina mothers’ breastfeeding experiences. Further investigation with larger samples of mothers living in other parts of the US is warranted to gain insights through each unique lens. Future work should continue examining breastfeeding through a multi-level lens and consider social determinants of breastfeeding (e.g., poverty) to enhance health equity for Latino families [30].

## Conclusion

To meet the goals of the Global Strategy on Infant and Young Child Feeding [1], the American Academy of Pediatrics [2], and Healthy People 2020 [10], breastfeeding promotion efforts should work to understand the context of breastfeeding for Latina mothers, as well as other racial/ethnic minority women. Considering the multilevel factors that shape Latina mothers’ breastfeeding is essential to develop and implement culturally tailored initiatives and facilitate access to breastfeeding support to improve maternal and infant health.

## Figures and Tables

**Table 1.** Sample interview questions

Construct	Questions
Importance of breastfeeding	In general, what are your views/perceptions about breastfeeding for the mother/for the child/for the relationship?
	What are your experiences around breastfeeding?
	What kind of challenges/difficulties have you experienced in breastfeeding?
Cultural differences between the US and native country	What would you do differently in terms of breastfeeding if you were in your native country?
	How would you characterize how people breastfeed in the US compared to your country?
	How do you think people would react to public breastfeeding in each of the two countries?
Social support for breastfeeding	How do you think your family (mother, father, etc.) perceives/supports breastfeeding?
	If you have friends/peers back in your native country, how would you characterize the way in which they perceive breastfeeding? If you have friends in the US, how would you characterize the way in which they perceive breastfeeding?
	How do healthcare services support or do not support breastfeeding?

**Table 2.** Demographic characteristics of Latina mothers (N = 9)

Participant	Nationality	Age	Number of Children	Breastfeeding Duration	Relationship Status	Education
Barbara	Brazil	32	1	Short	Married	Bachelor’s
Bianca	Brazil	43	2	Long	Married	Bachelor’s
Bruna	Brazil	42	2	Long	Married	Bachelor’s
Dolores	Dominican Republic	40	2	Short	In a relationship	Some college
Maria	Mexico	38	4	Varied	Married	Master’s
Maribel	Mexico	22	2	Long	Divorced	Less than high school
Mercedes	Mexico	36	2	Long	Married	Less than high school
Paloma	Puerto Rico	26	1	Short	Married	Bachelor’s
Patricia	Puerto Rico	49	2	Varied	Married	Bachelor’s
						

## Abbreviations:

US: United States
